# Optimizing Total Laparoscopic Hysterectomy for the Geriatric Population: A Practical Case Study and Comprehensive Literature Review

**DOI:** 10.7759/cureus.46265

**Published:** 2023-09-30

**Authors:** Vishal Bahall, Lance De Barry, Keevan Singh

**Affiliations:** 1 Department of Obstetrics and Gynaecology, The University of the West Indies, St. Augustine, TTO; 2 Department of Obstetrics and Gynaecology, San Fernando General Hospital, San Fernando, TTO; 3 Anaesthesia and Intensive Care Unit, Department of Clinical and Surgical Sciences, The University of the West Indies, San Fernando, TTO

**Keywords:** endometrial cancer, isolated tumor cells, laparoscopic surgery, gynaecology oncology, total laparoscopic hysterectomy (tlh), elderly individuals

## Abstract

Elderly patients represent a large cohort of patients requiring gynaecological surgery for benign and malignant indications. In recent years, several conventional gynaecological procedures have shifted towards minimally invasive alternatives such as laparoscopy, hysteroscopy, and robotic surgery. A recent Surveillance, Epidemiology, and End Results (SEER) analysis compared the outcomes of 25,000 women undergoing laparotomy versus laparoscopic approach to hysterectomy and found that laparoscopy is associated with a shorter duration of hospitalization (three days versus five days), less postoperative complications (76%), less requirement for blood transfusion, reduced operational costs, faster recovery, and an overall safer surgical experience. Although geriatric patients are affected by age-related comorbidities, physiologic changes, and altered pharmacodynamics and pharmacokinetics of administered drugs, these patients benefit most from minimally invasive surgery. In this paper, we present a comprehensive account of the interdisciplinary perioperative approaches employed to accomplish a total laparoscopic hysterectomy, bilateral salpingo-oophorectomy, and pelvic lymph node dissection in an 82-year-old patient who was diagnosed with grade I endometrial adenocarcinoma and multiple age-related medical comorbidities.

## Introduction

Over the span of a mere five years, from 2015 to 2020, a noteworthy transformation unfolded on a global scale. According to data from the World Health Organization (WHO), the proportion of individuals aged 60 years and over surged dramatically, leaping from 12% to a substantial 22% [1]. This demographic shift carries immense significance, demanding a re-evaluation of healthcare paradigms and a focused exploration of how best to cater to the evolving needs of our rapidly ageing world. As life expectancy increases, the number of patients presenting with surgically correctable gynaecological conditions is also expected to rise. In elderly patients, several factors conspire to present significant challenges in the perioperative period [2]. Compared to younger patients, elderly patients are more likely to have co-existing medical conditions, such as diabetes mellitus, hypertension, and heart disease, reduced major organ function, and a reduced functional reserve for invasive procedures [2]. While laparoscopic surgery has traditionally been considered a relative contraindication in elderly patients undergoing gynaecological surgery, it is plausible that the geriatric population stand to benefit the most from minimally invasive surgery [3].

Notably, there are several advantages to the laparoscopic approach to total hysterectomy in elderly patients. These include shortened recovery time, reduced duration of hospitalization, reduced postoperative pain and analgesia requirements, a lower incidence of postoperative complications, a faster return to routine activity, and a lower incidence of surgical site infections, venous thromboembolism, and wound dehiscence [4]. Moreover, there is a reduced risk of developing surgical site hernias, lower patient and institutional costs, and better cosmetic outcomes [4]. According to epidemiological data, several studies suggest that a minimally invasive approach to hysterectomy and uterine cancer staging significantly decreases morbidity in the immediate perioperative period in patients > 60 years old and that older women reported a higher quality of life score six months after total laparoscopic hysterectomy (TLH) [3].

In this regard, several measures exist to combat the technical and physiological drawbacks of TLH in the elderly population, and these include appropriate perioperative risk assessment, preoperative patient optimization, intraoperative technical considerations, and postoperative management strategies. Herein, we report a successful TLH, bilateral salpingo-oophorectomy, and pelvic lymph node dissection performed in an 82-year-old patient with grade I endometrial adenocarcinoma and several age-related comorbidities.

## Case presentation

An 82-year-old biparous female presented to the gynaecology clinic with complaints of postmenopausal bleeding for approximately two years. She described the bleeding as mild-to-moderate, associated with the passage of small clots and the use of about two to three sanitary pads per day. She denied experiencing abdominal pain, symptoms of anaemia, weight loss, reduced appetite, and urinary or gastrointestinal symptoms. She had a history of chronic hypertension and type II diabetes mellitus for approximately 24 years that was well-controlled and had no prior gynaecological or surgical history. Her medications included metformin 500 mg orally twice daily, nifedipine 20 mg orally once daily, and low-dose aspirin. She had no personal or familial history of malignancy.

On clinical examination, the patient appeared comfortable at rest and exhibited good body habitus for her age, with a BMI of 22.3 kg/m2. The abdominopelvic examination did not highlight any pelvic masses, pain, or tenderness. A speculum examination revealed a healthy-appearing cervix with no cervical masses, and she had a history of normal cervical cytology. A pelvic ultrasound scan highlighted an endometrial thickness of 10 mm and a 1.4 cm x 2.7 cm anterior subserosal uterine leiomyoma. There was no evidence of abdominopelvic free fluid, hydroureter, or hydronephrosis. Laboratory investigations highlighted microcytic anaemia (haemoglobin at 8.2 g/dL and mean corpuscular volume of 72.6 fL), glycated haemoglobin of 6.2%, and normal liver and renal function tests. An endometrial biopsy was performed and demonstrated grade I endometrial adenocarcinoma.

A computed tomography (CT) scan of the chest, abdomen, and pelvis highlighted a 12-week uterine size, a singular subserosal uterine leiomyoma measuring 1.5 cm x 2.7 cm, and an endometrial thickness of 10 mm. There was no evidence of pelvic or para-aortic lymphadenopathy, metastatic disease, or abdominopelvic free fluid. Treatment options were discussed with the patient and considering her age, motivation, and comorbidities, she consented to surgical staging that included a TLH, bilateral salpingo-oophorectomy, and bilateral pelvic lymph node dissection.

On preoperative assessment, her American Society of Anesthesiologists (ASA) score was 2 and her Eastern Cooperative Oncology Group (ECOG) score was 0. In preparation for surgery, aspirin was held for approximately five days prior to her procedure and upon admission, she was switched to an insulin sliding scale. Preoperative electrocardiogram and echocardiogram were both unremarkable and a chest X-ray highlighted no obvious pathology. Intraoperatively, the patient was placed in the dorsal lithotomy position at a 20-degree tilt with the legs secured in booted stirrups and a shoulder pad was placed to prevent displacement.

**Figure 1:**
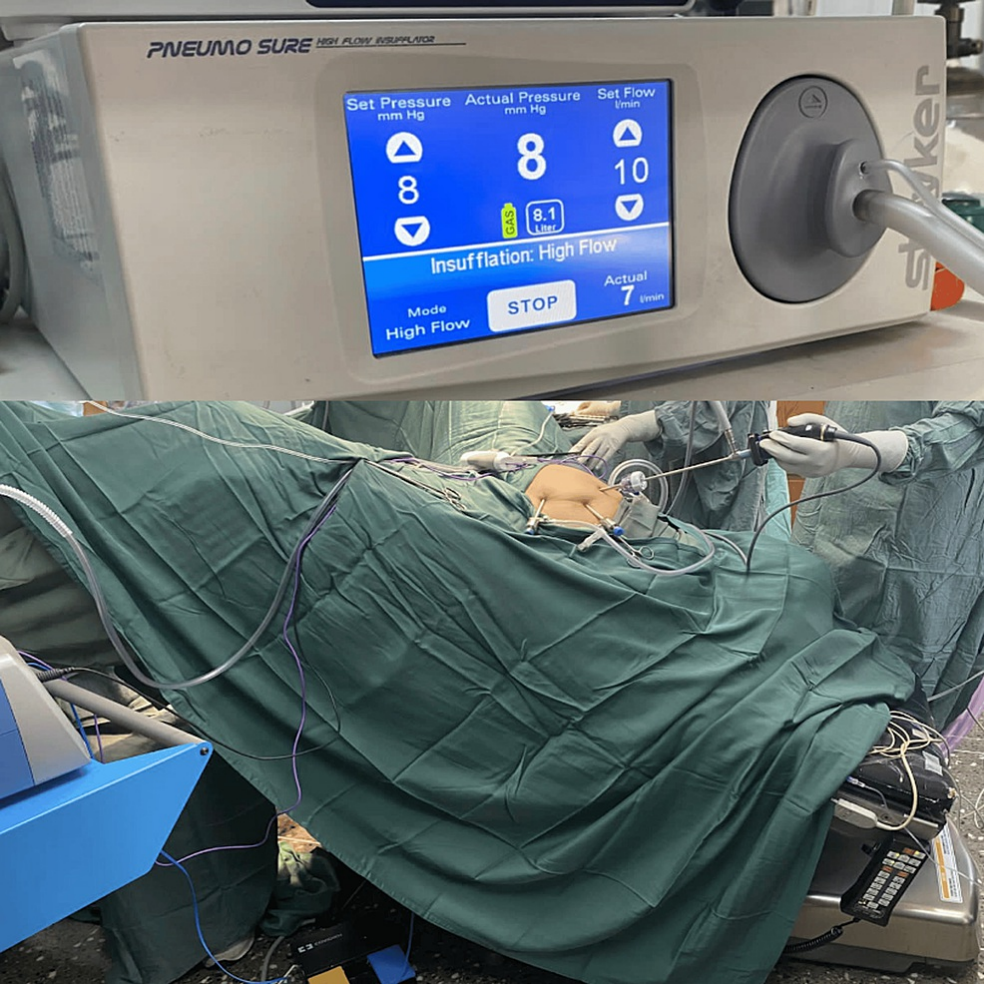
The patient was positioned in the Trendelenburg position at a 20-degree tilt. The pneumoperitoneum was maintained at a low operating pressure of 8 mmHg and a low flow rate of 10 L/min.

Under general anaesthesia with low airway pressure, a V-care uterine manipulator was placed. Abdominal entry was achieved with a 5 mm optical view trocar placed at the umbilicus at a 45-degree caudal entry angle to avoid injury to the abdominal aorta. The pneumoperitoneum was established at low flow (10 L/min) with a pressure of 10 mmHg. Two additional laparoscopic ports were placed in the left lower quadrant, lateral to the inferior epigastric vessels, and the operating pressure was maintained at 8 mmHg. The patient was placed in the Trendelenburg position at a 20-degree tilt and the standard procedure for TLH and bilateral salpingo-oophorectomy followed with particular attention directed to gentle tissue handling (Figure 1). The loose areolar tissue of the broad ligament was bluntly dissected using standard duck-billed laparoscopic grasper and LigaSure^TM^ (Medtronic, Dublin, Ireland). The uterovesical fold was carefully incised and caudal bladder deflection was meticulously performed to avoid inadvertent bladder injury. The colpotomy was performed utilizing the monopolar hook and upon the successful transvaginal evacuation of the specimen, an appropriately sized McCartney tube was placed in the vagina. The final step of surgery included the bilateral pelvic lymph node dissection. The retroperitoneal space was meticulously opened, and the loose areolar tissue was bluntly dissected. The external and internal iliac lymph nodes were carefully removed and placed in the McCartney tube for extraction. The obturator nerve was identified and the lymph nodes superior to this level were also sampled and extracted bilaterally. The vaginal vault was closed using a 2-0 delayed-absorbable barbed suture in a continuous fashion and all pedicles were thoroughly inspected for haemostasis. Throughout the procedure, low airway pressure was utilized. The total duration of the procedure was one hour and 15 minutes, and the estimated blood loss was approximately 150 ml.

Postoperatively, the patient was nursed in the cardiac position at 45 degrees. Her analgesic regime included intravenous and oral acetaminophen, parecoxib (a cyclo-oxygenase-2 (COX-2) selective non-steroidal anti-inflammatory drug (NSAID)), and a low dose of intramuscular morphine 5 mg every eight hours. To avoid postoperative delirium, the patient’s diet was advanced after six to eight hours, and the urinary catheter was discontinued after eight hours following satisfactory output. The patient was allowed uninterrupted night-time sleep and a close family member was allowed to stay with her. Mechanical thromboprophylaxis in the form of thromboembolic deterrent (TED) stockings was utilized and incentive spirometry was encouraged. Histopathology confirmed stage I endometrial adenocarcinoma that was deemed curative following her surgical staging. The patient’s postoperative course was unremarkable, and she was discharged in satisfactory condition two days later. On postoperative review at 10 days, six weeks, and three months later, she was well with no complaints.

## Discussion

Minimally invasive surgery is currently the standard of care for patients with endometrial cancer [5]. While the elderly population represents the fastest-growing cohort of patients worldwide, this patient population is increasingly requiring surgical intervention for both benign and malignant gynaecological conditions [4]. Age is an independent risk factor that ultimately leads to a higher risk of post-surgical complications; however, rejecting elderly patients for curative or palliative surgical procedures simply based on their age may deny them potentially life-saving procedures [6].

A recent Surveillance, Epidemiology, and End Results (SEER) analysis that examined approximately 25,000 women undergoing laparotomy for endometrial cancer found a higher incidence of postsurgical morbidity compared to laparoscopic surgery [7]. These included longer hospital stays (three days versus five days), increased postoperative medical complications (24%), and greater requirements for blood transfusion (6%) when compared to the laparoscopic approach [7]. Moreover, according to Scribner et al., among 125 patients with endometrial cancer, 13 patients over 77 years old successfully underwent a TLH, bilateral salpingo-oophorectomy, and pelvic lymph node dissection, and the conviction that advanced age is a contraindication to the laparoscopic procedure was disproved [8]. A prospective multicentre cohort study done by Briet et al. found that all features of quality of life significantly improved at six months postoperatively compared with preoperative values in patients older than 65 years of age undergoing TLH for both benign and malignant gynaecological conditions [3]. Following laparoscopic surgery, patients experienced less pain, a lower risk of wound complications, a shorter hospital stay, and faster recovery [3].

Patient selection and preoperative optimization are imperative to achieve safe surgical outcomes in elderly patients (Table 1) [9]. Most patients older than 65 years have comorbid conditions, including diabetes mellitus, hypertension, atherosclerosis, chronic obstructive pulmonary disease, and chronic kidney disease [2]. Preoperative risk assessment is particularly important to identify patients at high risk of adverse outcomes in the perioperative period [9]. Several scoring systems, such as the ASA score, the New York Heart Association (NYHA) classification, and the ECOG performance status, are utilized to identify high-risk patients [9]. Moreover, diabetes mellitus and hypertension should be tightly controlled to minimize perioperative risks and such patients should have a preoperative haemoglobin A1C (HbA1C) test, electrocardiogram, echocardiogram and medical review as necessary. Contraindications to laparoscopic surgery in elderly women are the same as for the general population and these include intestinal obstruction, a large tumour in the abdominal cavity, cardiac insufficiency, recent myocardial infarction, respiratory failure, severe obstructive lung disease, a history of multiple abdominal operations, and coagulation system disorders [10].

**Table 1:** Preoperative, intraoperative, and postoperative considerations for achieving successful laparoscopic hysterectomy in elderly patients. HbA1C: glycated haemoglobin; ASA: American Society of Anaesthesiologists Physical Status Classification; NYHA: New York Heart Association Functional Classification; ECOG: Eastern Cooperative Oncology Group Performance Classification.

Operative considerations	Features
Preoperative considerations	Laboratory investigations including complete blood count, kidney function test, HbA1C, electrocardiogram, chest X-ray, echocardiogram, and ASA, NYHA, and ECOG scoring systems
Intraoperative considerations	Proper positioning on the surgical table, utilizing uterine manipulator, maintaining pneumoperitoneum at a low pressure of 8 mmHg, utilizing a low flow rate of 10 litres/minute, and meticulous tissue handling
Postoperative considerations	Close postoperative monitoring, monitoring for delirium, multimodal approach to analgesia, venous thromboembolic prophylaxis, encourage early mobilization, and early discontinuation of the urinary catheter

TLH is performed in the dorsal lithotomy position or Trendelenburg position at a 20-degree tilt [11]. Proper positioning on the surgical table is important to prevent patient displacement, musculoskeletal injury, neurologic injury, and aggravation of pre-existing arthropathies [11]. Patients are secured in booted stirrups with liberal arm and leg padding to ensure proper positioning and padding of bony prominences in an attempt to maintain skin integrity and limit pressure on peripheral nerves [11]. Intraoperatively, abdominal entry and pneumoperitoneum are best achieved by utilizing an optical access trocar and a low insufflation pressure of up to 10 mmHg at a low flow rate [12]. Adjustments to minute ventilation will prevent deleterious effects related to lung compliance, functional residual capacity, and airway pressures [13]. Moreover, the risk of vascular and visceral injuries is significantly increased by utilizing traditional methods of achieving abdominal entry such as the Veress needle and Hasson technique [12].

In the elderly, tissue and vascular fragility is a major concern and meticulous tissue handling is required, particularly during bladder deflection and retroperitoneal dissection [14]. Furthermore, the placement of a uterine manipulator provides optimal exposition of the uterus during the procedure and the manipulator’s neck exposes the vaginal walls and enables safe incision with the monopolar electrode during colpotomy [15]. The vaginal vault is stapled or closed with delayed-absorbable barbed sutures and anchored to the uterosacral ligaments and cardinal ligaments to prevent vault prolapse. Finally, low intra-abdominal pressure should be utilized at 8-10 mmHg following port placement to support ventilatory efforts, improve intraoperative visualization, and significantly decrease the degree of postoperative pain experienced [14]. A study conducted by Albers et al. investigating the effect of low- versus normal-pressure pneumoperitoneum during laparoscopic surgery demonstrated lower pain scores, lower risk of shoulder tip pain, and faster recovery of bowel function postoperatively [16].

Postoperative pain is significantly less after TLH compared to abdominal hysterectomy [17]. Postoperative analgesia, including paracetamol, NSAIDs, tramadol, local anaesthetics, techniques such as patient-controlled analgesia, and strategies such as multimodal analgesia for acute pain management should be utilized in older patients [17]. However, the higher incidence of co-morbidities, concurrent use of other drugs, and pharmacokinetic and pharmacodynamic changes in elderly patients require careful titration of analgesics and an individualized approach to postoperative pain management. Furthermore, postoperative delirium is a quintessential geriatric complication with an incidence of 9-87% [18]. Recognition and treatment of postoperative delirium are critical to prevent functional decline, longer duration of hospitalization, institutionalization, and greater morbidity and mortality [18]. Factors contributing to the development of postoperative delirium include male sex, decreased fluid intake, multidrug therapy, prolonged use of Foley catheter, ICU admission, prolonged surgery duration, and certain medications [18]. Beers Criteria lists potentially inappropriate medications that should be avoided in elderly patients because they are ineffective or their risks of adverse effects such as delirium outweigh the benefits [19]. These medications include benzodiazepines, nonbenzodiazepine hypnotics, first-generation antihistamines, NSAIDs, and antispasmodics, among others [19]. Furthermore, early mobilization, diet advancement, incentive spirometry, and venous thromboembolism (VTE) prophylaxis are important to reduce perioperative VTE, postoperative delirium, and basal atelectasis leading to respiratory morbidity [18,20].

## Conclusions

In conclusion, laparoscopic surgery has become the treatment modality of choice for women requiring gynaecological surgery for certain benign or malignant pathology due to its proclivity for reduced perioperative complications and enhanced postoperative recovery. As the geriatric population increases worldwide, elderly patients are increasingly requiring gynaecological surgery for benign and malignant indications. Rejecting elderly patients for curative or palliative surgical procedures simply based on their age denies them potentially life-saving procedures. While laparoscopy was initially withheld from elderly patients due to concerns relating to its physiological demands, the elderly population stand to benefit the most from laparoscopic surgery and it should be offered unless specifically contraindicated. Laparoscopic surgery is associated with fewer perioperative complications, shorter duration of hospitalization, faster recovery, better cosmetic results, quicker return to normal activity, and lower patient and institutional costs.
